# Dynamic Thermal Management: Thermoelectric Vortices and Dynamic Tunable Magnetic Phase Transitions via Dynamic Chiral Thomson Effect on Rotating Conductors Exposed to Chopped Laser Beam

**DOI:** 10.3390/e28060661

**Published:** 2026-06-10

**Authors:** Gianpaolo Bei, Roberto Li Voti

**Affiliations:** Department of Basic and Applied Sciences for Engineering, Sapienza University of Rome, 00161 Rome, Italy

**Keywords:** out-of-equilibrium Barnett effect, quantized thermoelectric vortices, dynamic chiral Thomson effect, dynamic rotational Thomson voltage, dynamic tunable magnetic phase transitions, time-dependent Curie temperature, non linear chiral thermal emisssivity

## Abstract

In this work, we describe a new dynamic rotational Thomson effect induced on rotating conductors exposed to a chopped laser beam which generalizes recently observed analog magneto-transverse Thomson effects. We assume the existence of an out-of-equilibrium self-induced Barnett magnetic field that depends on helical thermal fields propagating on rotating conductors, and is associated with thermoelectric vortices. We deduce, assuming the validity of the Faraday law on the rotating out-of-equilibrium conductors, a time-dependent rotational Thomson voltage, showing that it is detectable on rotating ferromagnetic samples. We then prove the existence of dynamic tunable local magnetic phase transitions on rotating conductors associated with time-dependent Curie temperature fluctuations proportional to the dynamic Thomson voltage. Finally, we outline the relevance of this new time-dependent magneto-transverse Thomson effect either for dynamic thermal management or for dynamic tunable local insulator–metal transitions on rotating nanodisks exploiting metamaterials.

## 1. Introduction

Thermoelectric cooling is a research topic that has attracted significant attention in recent years, driven by the growing importance of solid-state cooling technology in developing more efficient thermoelectric modules in electronics and medicine.

Thermoelectric effects consist of the conversion of mutual heat into electricity and were discovered in 1821 by T.J. Seebeck, when he observed that two different metals at different temperatures generated an electric current at their two junctions. A few years later, in 1834, J.C.A. Peltier discovered the inverse effect, that is, a thermal gradient at the junction of two metals induced by an electric current. Based on these effects, W. Thomson predicted in 1851 a third thermoelectric effect, in which the cooling–heating effect depends on the sign of the thermal gradient along a single conductor carrying electric current [1].

It was then understood empirically that a good thermoelectric device should have low thermal conductivity k, high electric conductivity σ and a big Seebeck coefficient S to minimize irreversible heat dissipation due to the Joule effect and to preserve a big temperature gradient. Until the 1940s, thermoelectricity had been investigated only in metals, which had high thermal conductivity and therefore poor electrical performance and were not attractive for technological applications in thermoelectric coolers. In the late 1950s Joffe applied thermoelectricity to semiconductor samples and introduced the figure of merit [2]

$$zT=\frac{\sigma S^{2}}{k}$$

to measure its electric performance, proving that the maximum temperature difference ∆T was proportional to the figure of merit, dependent on $T_{c}$ being the cold junction of a Peltier cooler module:

$$∆T_{max}=\frac{zT_{C}^{2}}{2}$$

Despite the efforts of many researchers to improve the figure of merit, particularly at cryogenic temperatures, the levels of power generation and efficiency of the thermoelectric coolers were still very low relative to technological applications, and thermoelectricity was nearly abandoned. In the 1990s, green technology research stimulated renewed interest in thermoelectric coolers, due to the latter’s potential technological application to waste heat recycling. It has only been in recent years that Thomson cooler devices have been investigated, abandoning the conventional temperature-independent assumption exploited so far and extending the working range of Peltier coolers to larger temperature gradients and higher electrical currents [2]. Thermal diodes were then developed with temperature-dependent properties, observing metal–insulator phase transitions when the electric conductivity and the Seebeck coefficient had sharp changes. In fact, due to the Thomson effect, the heat density Q is proportional to both the electric current density $\vec{J}$ and the temperature gradient, by

$$Q=-\tau \vec{J}\cdot \nabla T$$

with ∇T the thermal gradient, τ the Thomson coefficient given by

$$\tau =T\frac{dS(T)}{dT}$$

and $S\left(T\right)=d∆V(T)/dT$ being the Seebeck coefficient associated with nonlinear heat transport.

This nonlinear contribution, due to the temperature-dependent Seebeck coefficient, implies an extra Joule–Thomson current in the heat diffusion equation, which modifies the efficiency of thermoelectric devices. This was evaluated, by most authors, assuming a constant Thomson coefficient, and exploiting unidimensional thermoelectric modules; the balance equation in the stationary approximation is given as

$$k\frac{d^{2}T}{dx^{2}}+\frac{J^{2}}{\sigma }-\tau J\frac{dT}{dx}=0,$$

neglecting, as conventionally assumed in the literature on thermoelectricity, stochastic fluctuations due to thermal noise and disorder effect.

A few years ago, in 2022, this new framework, based on the hypothesis of a constant Thomson coefficient, was applied successfully to magnetic phase transitions [3] and a giant Thomson coefficient τ≅−906 μV/K was detected at T = 305 K, using particular ferrite alloys. An enhanced Thomson effect with big temperature drops was then proven experimentally in 2023, measuring a steady temperature relative span ∆T/T≅5/38 [4]. This result greatly improved the performance of conventional thermoelectric coolers and was then confirmed, proving that an enhanced Thomson coefficient improves the efficiency of Thomson coolers in the presence of electronic and magnetic phase transitions.

Despite these impressive results obtained in the last three years and the efforts to improve Thomson cooler efficiency, the strategy to try to find materials to improve just the figure of merit by enhancing temperature gradients was not successful. In fact, these experimental investigations were not justified theoretically, since they assumed the existence of a figure of merit zT, the magnitude of which has been deduced so far using a stationary framework with a constant local equilibrium temperature T(x). As distinct from the conventional approach to thermoelectricity introduced originally by Joffe, it is not possible to define an equivalent definition of figure of merit zT for Thomson cooler devices [2] in a non-stationary framework, with a dynamic balance equation given by

$$\gamma \frac{dT}{dt}+div\vec{q}+w_{Joule}(T)+w_{TH}(T)+w_{\epsilon }(T)=0,$$

with $\frac{dT}{dt}$ a convective time derivative of a helical thermal field T propagating on the out-of-equilibrium rotating conductors. This generalized balance equation does not depend on the laser average power w, since it is assumed to be negligible far from the focus of an ultrathin laser, compared to the dynamic power associated to T comprising the rate of heat released in the unit of time caused by thermal emission $w_{\epsilon }\;(T)$, the rate of heat dissipated by the Joule effect in the unit of time $w_{Joule}\;(T)\;$ and the rate of heat released or absorbed in the unit of time due to the Thomson effect $w_{TH}\;(T)$.

Our work, in fact, is aimed at stimulating the thermoelectricity community to investigate new ways to improve the performance of thermoelectric Thomson coolers, overcoming the conventional stationary approach based on the figure of merit zT.

We will illustrate, therefore, a new dynamic approach to Thomson thermoelectric coolers, which, differing from the standard ones so far investigated, will have a complex-valued, thermal field-dependent magneto Seebeck coefficient S(T).

The main result of the framework proposed in this work is the prediction of a new dynamic chiral magneto thermoelectric effect, which we have called the dynamic rotational Thomson effect, and which consists of a pulsating Thomson voltage associated to chiral helicoidal thermal waves propagating on rotating conductors exposed to the harmonic heat source of the laser beam. We will show that the harmonic heat source of the chopped laser beam induces an out-of-equilibrium Barnett magnetic field, which allows the dynamic control of the direction and the intensity of the heat flux via a dynamic self-induced Righi–Leduc effect. This new dynamic chiral approach to thermoelectricity is nonlinear and non-stationary, and therefore, as distinct from conventional stationary approaches, predicts complex-valued time-dependent transport coefficients such as thermal conductivity, electric conductivity and Seebeck coefficient.

Therefore, it is not possible to evaluate the electric performance of the new rotating Thomson coolers by applying the conventional definition of the figure of merit zT, since it cannot be extended to non-stationary thermal gradients [5].

We will give some simple estimates of average Thomson voltages ∆V(T) associated with the dynamic non-linear Thomson cooling effect that are in accordance with recent experimental results measured on Thomson coolers associated to phase transitions [4]. We will show that on rotating ferromagnetic conductors, this effect can be exploited to implement dynamic tunable local magnetic phase transitions associated to detectable Curie temperature fluctuations.

We remark that this new time-dependent rotational approach to thermoelectricity, which depends on the thermal field T due to the harmonic heat source induced by the chopped laser beam, cannot be described by the standard stationary random fluctuations usually considered in complex systems with metastable states. In fact, the well-known Fluctuation–Dissipation Theorem [6], valid for stationary fluctuations, cannot be applied to out-of-equilibrium rotating metallic disks, nor can the Johnson–Nyquist theorem be deduced.

In fact, its thermal noise is usually assumed to be the white noise associated to a uniform stationary equilibrium temperature T

$$\sqrt{\bar{V^{2}}}=4k_{B}T\;R\;∆f$$

with $∆\bar{V^{2}}$ being the statistical variance of the voltage drops ∆V due to thermal fluctuations, $k_{B}$ the Boltzmann constant, R the constant resistance of the resistor of the Thevenin equivalent circuit, and ∆f the bandwidth of the noise.

Therefore, it is difficult to estimate the magnitude of the voltage perturbations due to statistical fluctuations on the new dynamic thermoelectric phenomena predicted by our model, since to deduce quantitative estimates, one would require a generalization of the Johnson–Nyquist theorem associated to a non-Gaussian time-dependent noise, such as Levy statistical noise caused by a non-Markovian process.

As, for example, the order of magnitude of the square root of the variance of the voltage fluctuations due to Johnson noise on a resistor with resistance of 1 kΩ is about 1 mV t, that is,

$$\sqrt{∆\bar{V^{2}}}\leq 10^{-6}\;V$$

We remark that the previous approach is not a realistic estimate of the effective resistances of Thomson thermoelectric devices, since usually, having at room temperature a resistance $R≅10^{-9}\;\Omega$, the phenomenon should produce the (very small) square root of the Johnson voltage variance

$$\sqrt{∆\bar{V^{2}}}\leq 10^{-12}\;V$$

making it negligible with respect to the average Thomson voltage ∆V(T), and this determination is predicted by our model to be of the order of millivolts.

We expect, therefore, that the predicted dynamic chiral thermal management effect is a stable effect with respect to thermal noise and that time-dependent non-Gaussian random fluctuations might not affect chiral symmetry, breaking transport effects induced on rotating conductors exposed to a chopped laser beam. An indirect experimental proof of our dynamic chiral Thomson effect could be given by measuring time-dependent violations of the Johnson noise on rotating nonlinear thermoelectric devices.

More generally, in accordance with recent investigations of the Fluctuation–Dissipation Theorem in the presence of external oscillating fields [7], we could interpret microscopically our chiral wavelike heat diffusion model as a non-Markovian heat transport process associated to non-Gaussian and non-stationary thermal noise with memory.

We remark that violations of the Fluctuation–Dissipation Theorem have been discovered recently in spin glass systems models [8] associated, as in our predicted thermomagnetic phase transitions, to the time-dependent local maximum of the specific magnetic entropy.

Despite these important results, investigations on the relevance of random fluctuations on the performance of thermoelectric devices are still missing, and it is impossible, as we will see in the Section 6, to estimate the magnitude of the thermal-noise-enhanced effect on rotating metallic samples with bidimensional stationary isothermal profiles T(x,y).

Moreover, in the modern literature on thermoelectric coolers, a discussion on the possible generalization of the Johnson–Nyquist Theorem on accelerated samples is missing, too [9], although a covariant generalization of this theorem could help to implement nano-Thomson coolers and to improve some experimental results involving the giant spin Thomson effect in semimetals, with strong spin–orbit coupling as bismuth.

In fact, although in the last ten years, the search for more efficient spintronic devices has stimulated some authors to investigate electric-field-induced orbital angular momentum in metals, a discussion on the relevance of a generalized Fluctuation–Dissipation Theorem is missing in the nascent research issue of non-equilibrium orbital physics called orbitronics [10]. One important research direction that has been explored very recently, using a stationary approach, assuming negligible perturbations due to non-Gaussian thermal-noise effects, is how to convert orbital currents to charge currents to enhance the spin Seebeck effect and improve the electric performance of thermoelectric devices. This new research issue of orbital physics, although currently lacking a solid experimental verification of the predicted orbital currents, might explain new phenomena such as the orbital Hall effect and the anomalous Hall effect in ferromagnets and semimetals [1]. These studies have not previously investigated the effects of out-of-equilibrium orbital currents on enhancing the efficiency of junction-less thermoelectric devices, which, conventionally, exploit the classical stationary Thomson effect or its phase-transition-induced enhancement [2,11,12].

Our work, on the contrary, is aimed at developing an out-of-equilibrium generalization of the Thomson effect on macroscopic rotating metals and semimetals, with the Thomson coefficient dependent on the thermal waves propagating on the moving samples [13,14], an approach which might be exploited to implement dynamic rotational Thomson thermoelectric coolers. The principal motivation that inspired our proposal is to elaborate a new unified framework of heat diffusion and thermal emission based on orbital physics that might implement chiral thermal management and tunable magnetic phase transitions via a dynamic chiral Thomson effect.

The theoretical proposal we will illustrate in the following sections develops a novel dynamic rotational chiral approach to thermoelectricity and thermal emission based on a new, generalized magneto-chiral Thomson effect. When confirmed experimentally, the stability of this chiral symmetry-breaking effect with respect to stochastic fluctuations due to thermal noise and disorder effects might be exploited for technological applications such as chiral thermal management, non-reciprocal heat transport and non-reciprocal chiral photonics. Therefore, as we will explain in Section 6, the predicted dynamic chiral magneto Thomson cooling-heating effect could be enhanced by non-Gaussian Levy stochastic fluctuations, paving the way for dynamic extension of the noise-enhanced effect on wavelike heat diffusion process, which is different from soliton propagation in quantum Josephson junctions.

This broader physical framework based on statistical fluctuations might affect, we think, the deterministic stationary approach used so far in recent experiments confirming the existence of the magneto Thomson effect and of the transverse Thomson effect [15,16]. On the contrary, as we will show in the following, the stability and the observability of our dynamical chiral Thomson effect, depending on the convective time derivative of the out-of-equilibrium Barnett magnetic field ∆B(T), will not be affected by the presence of a time-dependent Poisson-like thermal noise, since this random fluctuation has not so far been analyzed on rotating samples with a dynamic chiral symmetry-breaking effect.

We will show, in fact, that the harmonic heat source due to a chopped laser beam induces a dynamic enhancement of the Thomson coefficient τ(T), giving an average detectable estimate of this giant chiral thermoelectric effect in rotating iron samples.

Finally, we will deduce the existence of a chiral tunable thermal emissivity associated to the gauge-breaking dynamic Thomson voltages, discussing its relevance for a future nonlinear approach to thermal harvesting [17], chiral thermal emission, and nonreciprocal photonics [18,19] on rotating conductors.

## 2. Out-of-Equilibrium Barnett Effect, Dynamic Thermal Management and Irrotational Thermoelectric Fields

The theoretical proposal we will illustrate in this section develops a novel dynamic rotational approach to thermal conduction based on out-of-equilibrium generalization of the Barnett effect associated to an effective magnetic field $\vec{∆B(T)}$ dependent on helical thermal fields generated on rotating metallic disks. We remark, as illustrated in the Introduction, that although this magnetic field might depend on time-dependent stochastic fluctuations, it is robust and detectable with respect to conventional stationary thermal-noise effects.

We will assume that the harmonic heat source due to the chopped laser beam on the rotating disk induces an effective temperature-dependent Barnett magnetic field $\vec{∆B(T)}$ parallel to the angular velocity vector of the body $\vec{\Omega }$ [20,21], that is, an out-of-equilibrium thermomagnetic effect:

(1) $$\vec{∆B(T)}=\frac{\vec{\Omega }}{g(T)}=\frac{2m(T)}{e}\;\vec{\Omega },$$

with e the electron charge, g(T) the out-of-equilibrium electron gyromagnetic ratio, m(T) the effective electron mass and $\vec{∆B(T)}$ the out-of-equilibrium Barnett magnetic field perturbation, measured with respect to the average vertical component of Earth’s magnetic field $B_{0}≅45\;mT.$

This effective magnetic field induced on rotating conductors by a chopped laser beam depends on a time-dependent thermal field T, which is assumed to be an harmonic solution of a telegraphist equation associated to a wavelike non-linear heat diffusion law (Appendix A)

(2) $${D_{t}}^{2}T+\frac{D_{t}T}{\tau \left(r,\omega^{\prime }\right)}-{v_{T}}^{2}\nabla^{2}T=0,$$

with $D_{t}=\frac{\partial }{\partial t}+\Omega \cdot \frac{\partial }{\partial \theta }$ a convective time derivative solidary to the rotating disk, ${v_{T}}^{2}$ the square of the conventional thermal wave phase velocity and T the chiral helical thermal fields associated to the fluctuation of equilibrium temperature of the sample $T_{0}$

(3) $$T\left(r,\theta ,t\right)=T_{0}e^{i\left[\beta \left(r\right)r+m\theta -\omega^{\prime }t\right]},$$

and $\beta \left(r\right)r+m\theta -\omega^{\prime }t=\varphi (r,\theta ,t)$ the space time-dependent phase of the helical thermal field T.

This new dynamic approach to magnetic phase transitions via a time-dependent self-induced Barnett magnetic field depends on the existence of a new time thermal-field-dependent function b(T), so that Equation (1) can be rewritten as

(4) $$\vec{∆B(T)}=\frac{2m\left(T\right)\vec{\Omega }}{e}=\frac{b(T)\vec{\Omega }}{A},$$

with A an average of the unknown Righi–Leduc coefficient A(T) with respect to T, dependent on the metal sample considered (Appendix A).

In fact, the out-of-equilibrium Barnett field makes irrotational and anisotropic the heat flux density $\vec{q\left(T\right)}$, due to the presence of an additive transverse heat flux term $\vec{q\left(T,\vec{\Omega }\right)}$ due to the dynamic Righi–Leduc effect $b(T)\vec{\Omega }\times \nabla T$ [14].

Therefore, assuming the validity of the Wiedermann–Franz law, the non-linear magnetic-controlled heat transport process must be must be associated to an out-of-equilibrium irrotational thermoelectric field

(5) $$\vec{E_{T}}=-S\left(T\right)\nabla T,$$

with S(T) a time-dependent complex-valued magneto Seebeck coefficient.

Assuming on the rotating out-of-equilibrium conductors a generalized out-of-equilibrium Faraday–Maxwell induction law

(6) $$rot\;S\left(T\right)\nabla T=\frac{d\vec{∆B\left(T\right)}}{dt}=\frac{db\left(T\right)}{dt}\vec{\Omega }=\frac{dm(T)}{edT}\frac{dT}{dt}\vec{\Omega }$$

that shows that the thermoelectric field is irrotational whenever it is present, a thermal field propagates on the rotating disk. We note that by finding approximate solutions to Equation (6) it is possible to deduce estimates of the electron effective mass m(T) and its dependence on the dynamic magneto Seebeck coefficient S(T). For example, assuming that the rotor of the thermoelectric field of Equation (5) is proportional to it, it is easy to show that S$\left(T\right)\propto \Omega \frac{dm(T)}{edT}$, showing that a constant m(T) makes S(T) equal to zero.

The non-linear and transverse heat flux law due to the chopped laser beam incident on the rotating conductors can be described, neglecting thermal effects due to phonon scattering, by a wavelike model (Appendix A), which is in accord with a gauge-symmetric approach to wavelike heat conduction recently investigated [22].

In fact, the out-of-equilibrium Barnett magnetic field of Equation (1) induces, via a dynamic, self-induced Righi–Leduc effect, a transverse heat flux [15], which makes heat transport a non-linear transverse anisotropic process described by

(7) $$\vec{q\left(t+\tau \right)}=-k\nabla T+b(T)\vec{\Omega }\times \nabla T,$$

with τ the local non-linear electron relaxation time of the rotating conductor, which can be determined explicitly (Appendix A), k the standard thermal conductivity of the conductor at rest and T the harmonic helical thermal field.

Since the wavelike non-linear heat flux of Equation (7) is irrotational, that is, $rot\vec{q\left(t\right)}\neq 0$, it implements, due to the linear dependence on the angular velocity vector $\vec{\Omega }$ of the rotating conductor, a new dynamic chiral approach to thermal management. The heat transfer is controlled by the transverse thermoelectric current density associated to the transverse thermoelectric field introduced in $\vec{E_{T}}$ Equation (5), and is defined, assuming the Weidermann–Franz law, on the rotating conductors by

(8) $$\vec{J_{T}}=\sigma \vec{E_{T}}=S_{0}\sigma \left(T\right)\nabla T=\frac{S_{0}}{L}k(T)\nabla lnT,$$

with $S_{0}$ an average Seebeck coefficient, σ(T) an effective time-dependent electric conductivity, L the Lorenz number and k(T) an effective thermal conductivity. The irrotational thermoelectric density vector $\vec{J_{T}}$ can be associated to topologically stable thermoelectric vortices $\oint \vec{J_{T}}\cdot d\vec{r}$ propagating on the rotating conductors, which, in the simple case of a linear dependent Seebeck coefficient S(T), are proportional to the phase change on the isothermal profile circuit of lnT.

We note that this new wavelike non-linear heat diffusion model is a generalization of the Cattaneo–Vernotte model [23,24] that, as we will see in the next paragraph, can be associated to a robust and stable dynamic rotational Thomson effect.

## 3. Dynamic Chiral Thomson Effect and Tunable Magnetic Phase Transitions on Rotating Metallic Disks

We will show in this section that the Barnett magnetic field self-induced by rotation ∆B(T) of Equation (1), which is parallel to the axis of symmetry of the rotating disks, will generate a dynamic Thomson voltage ∆V(T,Ω) [14], the radial pulsating electric field of which will tend to counteract the dissipative heating process occurring due to the Joule effect.

In fact, according to Faraday’s law, an out-of-equilibrium oscillating electromotive force $∆V\left(T,\Omega \right)$ proportional to the time derivative of the Barnett magnetic field of Equation (1) is induced on the rotating disk, and is proportional to the angular velocity Ω of the rotating conductor

(9) $$∆V\left(T(r),\Omega \right)=-\frac{d\varphi (∆B\left(T\right))}{dt}=-\int_{0}^{r}\Omega \frac{db(T)}{dt}2\pi rdr,$$

which, by using the Zel’dovich condition (Appendix A) and the function b(T) introduced in Equation (2), can be shown to be proportional to the angular velocity of the rotating disk Ω

(10) $$∆V\left(T,\Omega \right)=i\frac{\omega^{\prime }\Omega }{A}\int_{0}^{r}\frac{db(T)}{dT}T2\pi rdr,$$

with $\omega^{\prime }$ the shifted pulsation of the thermal field due to the Zel’dovich rotational super radiance effect [25,26], (Appendix A)

(11) $$\omega^{\prime }=\omega -m\Omega ,$$

with ω the pulsation of the chopper, Ω the angular velocity of the rotating disk and m an integer number associated to the topological index of the helical thermal fields T.

In fact, it can be shown that, assuming a non-Fourier wavelike heat diffusion model [27] with convective time derivative, there are harmonic helical thermal fields T propagating on the rotating disk, which is given by (Appendix A)

(12) $$D_{t}T=\left(\frac{\partial }{\partial t}+\Omega \cdot \frac{\partial }{\partial \theta }\right)T=-i\omega^{\prime }T,$$

This dynamic Thomson voltage is associated to an oscillating chiral radial Thomson electric field $\vec{E}$ (T, Ω) tuned by Ω, with a radial component given by

(13) $$E_{r}\left(T,\Omega \right)=-\nabla_{r}∆V\left(T,\Omega \right)=-\frac{d∆V\left(T,\Omega \right)}{dr}=i2\pi r\frac{\omega^{\prime }\Omega }{e}\frac{dm\left(T\right)}{dT}T,$$

which is proportional to the dynamic magneto Seebeck coefficient S(T) of Equation (5), since $\frac{d∆V\left(T,\Omega \right)}{dr}=S(T)\frac{dT}{dr}$.

This new dynamic chiral Thomson electric field might be used to improve the electric performance of Thomson cooler devices, exploiting thermally driven magnetic phase transitions. In fact, the radial component of the time-dependent Thomson electric field $E_{r}\left(T,\Omega \right)$ depends on the time derivative of the Barnett magnetic field and can be enhanced, tuning by Equation (13), the shifted pulsation $\omega^{\prime }$ of the thermal field and the angular velocity Ω of the rotating disk.

This time-dependent radial thermoelectric field dynamically perturbs electron conduction bands, making Fermi energy time-dependent, pushing electrons harmonically outward and inward with respect to the center of the rotating disk, in accordance with a generalization of the Stewart–Tolman effect recently investigated by some authors interested in general approaches to out-of-equilibrium thermodynamics of neutron stars [28,29,30].

**Figure 1:**
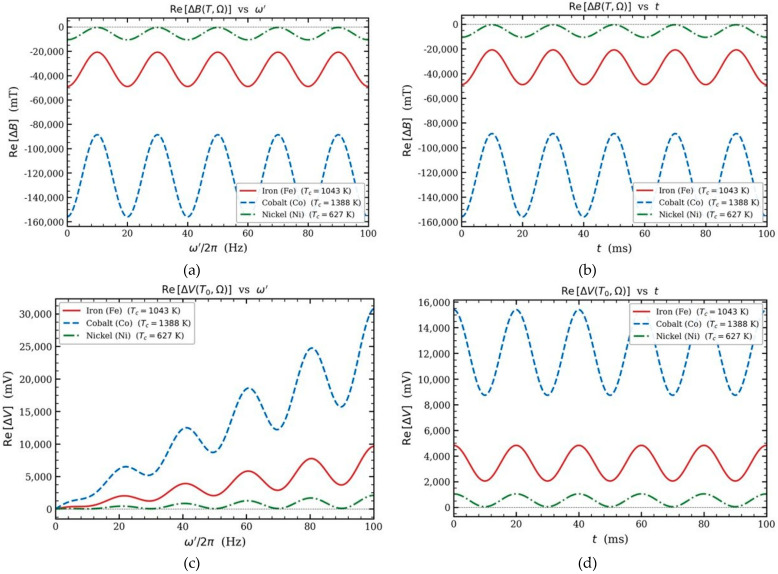
**Dependence of the real part of the dynamic Barnet magnetic field and of the dynamic rotational Thomson voltage of iron, nickel and cobalt on the shifted pulsation and as to time with** $B_{0}\;=\;45\;mT,R\;=\;0.01\;m,T_{0}\;=\;300\;K$**.** (**a**) plot of the harmonic oscillations of the real part of the Barnett magnetic field with respect to the shifted pulsation $\omega^{\prime }$ of Equation (11) in Herz; (**b**) plot of the harmonic oscillations of the real part of the Barnett magnetic field with respect to time t; (**c**) plot of the dependence of real part of the dynamic rotational Thomson voltage on the shifted pulsation $\omega^{\prime }$ of Equation (11) in Herz; (**d**) plot of dependence of the real part of the dynamic rotational Thomson voltage on the time t.

As a case study of the rotation-induced thermoelectric effect associated to magnetic phase transitions, we illustrate some simple estimates of the average dynamic Thomson voltage $∆V\left(T,\Omega \right)$ of (28) in the simple case of ferromagnetic disks. We will introduce a generalized Curie–Weiss-like magnetization law associated to the out-of-equilibrium Barnett magnetic field of (1), assuming as first approximation a time-dependent Curie–Weiss-like law for the paramagnetic state of the out-of-equilibrium rotating ferromagnetic disks (Figure 1)

(14) $$∆B\left(T,\Omega \right)=B_{0}T_{c}\frac{\chi }{\left(T-T_{c}\right)}$$

with the χ the average constant real part of the magnetic susceptivity, $B_{0}$ the constant average vertical component of the environment magnetic field chosen in the experimental setup and $T_{c}$ the constant and uniform Curie temperature of the metallic sample at rest.

Taking the real part of the Barnett field as the first member of Equation (14), we get a harmonic time-dependent magnetization in the paramagnetic state of the rotating conductor:

(15) $$Re∆B\left(T_{0},\Omega \right)=\frac{\chi T_{c}B_{0}}{T_{0}\;(1-cos\omega^{\prime }t){-T}_{c}}$$

Inserting the relation in Equation (13) into Equation (9) we can deduce an estimate of the real part of the oscillating rotational Thomson voltage self-induced on the border of the rotating disk in the paramagnetic state:

(16) $$Re∆V\left(T_{0},\Omega \right)≅-\frac{\pi (\omega^{\prime }sin\omega^{\prime }t)\chi B_{0}T_{0}{T_{c}R}^{2}}{{\left[T_{0}\;(1-cos\omega^{\prime }t){-T}_{c}\right]}^{2}}≅-\frac{\pi \omega^{\prime }\chi^{\prime }B_{0}{T_{c}R}^{2}}{T_{0}\;(1-cos\omega^{\prime }t){-T}_{c}}≅-\frac{\pi \omega^{\prime }\chi B_{0}{T_{c}R}^{2}}{T_{0}\;(1-cos\omega^{\prime }t){-T}_{c}}$$

with $\chi ’≅\frac{\chi sin\omega^{\prime }tT_{0}}{T_{0}\;(1-cos\omega^{\prime }t){-T}_{c}}≅\chi$ an effective time-dependent magnetic susceptivity on the rotating disk, assumed, as a first order approximation, to be equal to the constant average real part of the magnetic susceptivity χ of the sample at rest.

We reported in Figure 1 the plots for the three ferromagnetic conductors iron, nickel and cobalt as a function of the shifted pulsation in Herz $\frac{\omega^{\prime }}{2\pi }$ of Equation (11) and as function of time, either of the real part of the Barnett magnetic field or that of the real part of the self-induced dynamic rotational Thomson voltage of Equations (15) and (16).

We remark that the predicted giant peaks of the real part of the rotational Thomson voltage ∆V(T) of Figure 1c could be detected as giant magnetoresistance resonances associated to magnetic and thermal phase transitions. The order of magnitude of these dynamic magnetoresistance resonances could be estimated by inserting in Equation (16) a complex-valued thermal field-dependent magnetic susceptivity and Curie temperature and imposing that the dynamic Thomson electric power is equal to the Joule dissipative power.

Anyway, we expect, as we described in the introduction, that the dynamic rotational Thomson effect is stable with respect to the thermal-noise effects and disorder effects, since $∆B\left(T,\Omega \right)$ in Equation (1) due to rotation-induced stable thermoelectric vortices associated to the irrotational thermoelectric current of Equation (8). It is possible to improve the estimate by solving the recursive differential equation obtained by substituting in Equation (14), for the constant Curie temperature $T_{c}$ the dynamic one $T_{c}^{\prime }(∆V)$ given by

(17) $$T_{c}^{\prime }\left(∆V\right)=T_{c}+∆T_{c}(∆V)≅T_{c}+\frac{Re∆V(T)}{ReS(T)},$$

and getting a second order approximation of the recursive equation

(18) $$Re∆V\left(T_{0},\Omega \right)≅-\frac{\pi \omega^{\prime }\chi B_{0}{T_{c}R}^{2}}{T_{0}\;\left(1-cos\omega^{\prime }t\right)-T_{c}^{\prime }\left(∆V\right)}≅≅-\frac{\pi \omega^{\prime }\chi B_{0}{T_{c}R}^{2}}{T_{0}\;\left(1-cos\omega^{\prime }t\right)-\frac{Re∆V}{S_{0}}},$$

with $S_{0}$ the average Seebeck coefficient of the ferromagnetic sample at rest.

Therefore, we have proven that that by assuming a Barnett magnetic field in the paramagnetic state given by Equation (14) the harmonic heat source of a chopped laser beam induces on rotating ferromagnetic disks tunable magnetic phase transitions associated to time-dependent Curie temperature fluctuations that, by Equation (17), can be estimated to be on the order of magnitude of 100 Kelvin.

This prediction could be tested in laboratories with modern infrared thermocameras, applying lock-in techniques, tuning the physical constants in Equation (16) so to detect peaks of the average rotation-induced chiral Thomson voltage or of the average of the oscillating magnetic susceptivity.

We will study the stability of the effects from a general out-of-equilibrium thermodynamic approach, assuming that dynamic magnetic phase transitions induced on the rotating conductors are associated to non-stationary local equilibrium states predicted by the general principle of equilibrium of maximum local entropy rate production.

In fact, the new oscillating thermoelectric field dependent $\vec{E_{T}}$ of Equation (5) dependent on the dynamic magneto Seebeck coefficient S(T) can be associated to an out-of-equilibrium thermodynamic process with specific entropy production in a rotating frame which generalizes the conventional one on a sample at rest [7]

(19) $$\partial_{t}s\left(T,\Omega \right)=Re\frac{div\vec{q}}{T},$$

(20) $$D_{t}s\left(T,\Omega \right)=Re(\frac{{\vec{q}}^{2}T}{\sigma }+k\left({\nabla T}^{2}\right)-i\omega^{\prime }\gamma T),$$

with σ the electric conductivity.

We note that our model, as distinct from a similar wavelike nonlinear heat diffusion model recently investigated [31], depends on the dynamic chiral Thomson effect previously discussed by

(21) $$\vec{q(t+\tau )}=S(T)T\vec{J}-k\nabla T,$$

with S(T) a dynamic generalization of the magneto Seebeck coefficient recently investigated [32].

From Equation (6) we deduce a relation that will allow us to express the dynamic magneto Seebeck coefficient as a function of the free parameter b(T) introduced in Equation (1):

(22) $$S\left(T\right)T\vec{J}=A\left[\vec{∆B\left(T,\vec{\Omega }\right)}\times \nabla T=b(T)\vec{\Omega }\times \nabla T\right],$$

with the orbital electric current density $\vec{J}$ given by

(23) $$\vec{J}=\sigma \left(\vec{E-}S\nabla T\right)$$

We note that the specific entropy production of Equation (19) can be negative whenever the gradient term due to the Thomson effect is bigger than the first term due to the Joule effect. Moreover Equation (19) can be exploited to dynamically tune magnetic phase transitions, assuming that they are associated to the maxima or minima of the entropy flux rate comoving with the rotating disks, that is,

(24) $$D_{t}s\left(T,\Omega \right)=\frac{\partial }{\partial t}s\left(T,\Omega \right)+\Omega \cdot \frac{\partial }{\partial \theta }s\left(T,\Omega \right)=0,$$

using the time convective derivative $D_{t}$ solidal to the rotating disks of Equation (11).

From Equation (22) we can generalize the stationary local conservation law of energy density and the conventional specific entropy flux rate of a system at rest [12], taking into account that it depends on the helical thermal fields solutions of the telegraphist equation introduced in Equation (16):

(25) $$D_{t}s=\frac{\vec{E}\;\cdot \vec{J}}{T}-\frac{div\vec{q}}{T}=\frac{{\vec{J}}^{2}}{\sigma T}+k\frac{{\left(\nabla T\right)}^{2}}{T^{2}}=\gamma D_{t}T=-i\gamma \omega^{\prime }T,$$

By Equations (19) and (23), it follows that the electric current density on the thermal field T satisfies the relation

(26) $${\vec{J}}^{2}=-\sigma (k{\nabla lnT}^{2}+i\gamma \omega^{\prime }T).$$

This equation is the stability condition which must be satisfied to have a dynamic chiral Thomson effect on rotating conductors robust with respect to random fluctuations and disorder effects, which usually are associated in the literature with the random magnetic fluctuations described by Gaussian white noise.

We remark that by inserting the second member of Equation (23) into Equation (26), the explicit dependence on the thermal field T of the dynamic magneto Seebeck coefficient S(T) can be deduced, making it possible to compare the order of magnitude of this effect with the stationary estimates associated to Joule dissipative noise.

Equation (26) shows, therefore, that the dynamic chiral Thomson effect is a robust, experimentally detectable effect that can be exploited on rotating conductors exposed to chopper laser beams, allowing coherent control by thermal fields of magnetization in ferromagnetic samples, tuning their dynamically local magnetic phase transitions. Therefore, the predicted Curie temperature harmonic fluctuations could be easily detected in laboratories with modern infrared thermocameras (Flir, Portland, OR, USA) applying lock-in techniques and could be exploited as signatures of giant magnetic phase transitions on rotating ferromagnets and of thermal phase transitions on rotating metamaterial disks such as those incorporating vanadium dioxide.

We note that the laser-induced out-of-equilibrium thermodynamics on rotating metallic disks implies, taking in account the dynamic chiral Thomson effect, the following generalization of the Faraday law on the rotating metallic disk given by

(27) $$rot\vec{(E}-S(T)\nabla T)=-D_{t}\vec{∆B(T)},$$

with $\vec{∆B(T)}$ the out-of-equilibrium Barnett magnetic field of Equation (1).

This equation implies that, by Equation (11), due to the Zel’dovich super radiant effect, that rotation induces on out-of-equilibrium conductors topological stable chiral thermoelectric vortices. Independently from thermal-noise effects and disorder effects, they can be detected experimentally by observing a change of sign of the rotor of dynamic Thomson electric field of Equation (5), since we have

(28) $$rot\;S(T)\nabla T=\frac{d}{dT}\vec{B\left(T\right)}\;\frac{dT}{Dt}=i\omega^{\prime }T\frac{d}{dT}\vec{B\left(T\right)},$$

whenever the shifted pulsation $\omega^{\prime }$ is non-zero.

Therefore, our model predicts the existence of a time-dependent irrotational magneto Thomson effect that is stable and robust with respect to random fluctuations, which generalizes recently observed magneto and transverse Thomson effects [2,10], whenever the shifted pulsation $\omega^{\prime }$ is non-zero.

We remark that the predicted dynamic chiral magneto Thomson effect allows us to deduce, once Equation (26) is solved, the dynamic magneto Seebeck coefficient S(T), and hence the thermal field dependence of the Thomson coefficient [12],

(29) $$\tau_{TH}(T)=T\frac{dS(T)}{dT},$$

then allowing us to deduce estimates of the performance of this non-linear time-dependent thermoelectric effect.

Finally, we note that the Zel’dovich rotational super radiant effect could be exploited to improve the electric performance of a dynamic Thomson cooler, making the thermoelectric field of (5) reduce the relative magnitude of the Joule heating process for the effective thermal field dependent impedance of the disk Z(T).

## 4. Dynamic Rotational Thomson Effect and Gauge-Breaking Chiral Thermal Emission

We showed in the previous section that the oscillating chiral Thomson voltage, introduced in Equation (16) for an iron disk, depends on the sign of the rotational Doppler shift $\omega^{\prime }$ of thermal field T. Therefore, since its time derivative is proportional to the thermal power density, it can be associated to the chiral thermal radiation emitted by the out-of-equilibrium rotating conductors.

In fact, in accordance with the new, recent approach to non-reciprocal photonics and tunable thermal emissivity on metamaterials [18,33], assuming the Stefan–Boltzmann law, a chiral dynamic tunable thermal emissivity dependent on the out-of-equilibrium Barnett magnetic field can be introduced:

(30) $$e\left(∆B(T),\Omega \right)=\alpha Re\frac{div\;{\vec{P}}_{T}(T,\Omega )}{\sigma \left(T^{4}-T_{0}^{4}\right)},$$

with σ the Stefan-Boltzmann constant, α a dimensional constant dependent on the disk sample and ${\vec{P}}_{T}(T,\Omega )$ an irrotational chiral thermal Poynting vector proportional to the dynamic magneto Seebeck coefficient S(T)

(31) $${\vec{P}}_{T}(T,\Omega )=\frac{-S(T)\nabla T\times ∆\vec{B(T)}}{\mu },$$

with $rot\;{\vec{P}}_{T}(T,\Omega )\neq 0$

Using Equation (4), this can be rewritten, making explicit the linear dependence on the angular velocity Ω of the Poynting vector:

(32) $${\vec{P}}_{T}\left(T,\Omega \right)=\frac{b(T)S(T)\vec{\Omega }\times \nabla T}{\mu },$$

proving that the dynamic chiral Thomson effect is associated to chiral thermal remission process.

Equation (32) implies that the dynamic chiral thermal emissivity of Equation (30) implements the chiral polarized thermal radiation emitted by rotating conductors exposed to a chopped laser beam, which could be detected when looking for a dynamic nonlinear magneto Kerr effect.

We can introduce an out-of-equilibrium magnetic vector potential $\vec{A_{T}(T)}$ the rotor of which can be associated to the out-of-equilibrium Barnett magnetic field

(33) $$rot\vec{A_{T}\left(T\right)}=\vec{∆B(T)},$$

by the conventional definition

(34) $$\frac{d\vec{A_{T}\left(T\right)}}{dt}=S(T)\nabla T$$

which breaks gauge invariance, since it satisfies the relation

(35) $$c^{2}div\vec{A_{T}\left(T\right)}+\partial_{t}∆V\left(T\right)=-(\Omega \cdot \frac{\partial }{\partial \theta })∆V\left(T\right),$$

which can be approximated as

(36) $$c^{2}div\vec{A_{T}\left(T\right)}+\partial_{t}∆V\left(T\right)≅-\Omega S\left(T\right)∆T$$

Introducing a path integral on a closed circuit of the time derivative of the out-of-equilibrium magnetic field as the first member of Equation (34), the existence of chiral electromagnetic radiation emitted by the rotating disks can be deduced, since

(37) $$\oint \frac{d\vec{A_{T}\left(T\right)}}{dt}\cdot \vec{dl}=\oint S(T)\nabla T\cdot \vec{dl}$$

Associated to topological stable chiral electromagnetic vortices, this is

(38) $$\oint \vec{A_{T}\left(T\right)}\cdot \vec{dl}\neq 0$$

We note that the gauge-breaking dynamic rotational Thomson voltage is a gauge-breaking field that can be exploited, using Equation (36), to enhance the performance of Thomson coolers and heating devices, and when confirmed experimentally, could be an indirect test of recent applications of Extended Electrodynamics theory to thermally induced gauge-breaking effects and relevant to recent investigations of magnetic field-driven thermal management [34,35,36,37,38].

We hope that our theoretical proposal will stimulate the thermoelectricity community to investigate the relevance of the gauge-breaking dynamic magneto Thomson effect to understand out-of-equilibrium thermodynamics processes and anisotropic magnetization processes, either for rotating nanodisks or for fast-rotating macroscopic systems such as neutron stars [29,30].

## 5. Discussion

The main motivation which inspired our novel proposal of dynamic rotating thermoelectric coolers is to elaborate a new unified framework of anomalous heat diffusion, tunable chiral thermal emission and dynamical orbital physics which might have innovative technological applications in the future such as chiral tunable thermal diodes and heat-assisted dynamic magnetic recording.

We illustrated a new dynamic chiral approach to Thomson electric coolers, which, as distinct from the conventional stationary approaches so far investigated, predicts a complex-valued time-dependent Seebeck coefficient and Thomson coefficient. Therefore, in our dynamical non-linear approach to thermoelectricity, it is not possible to use standard notions of electric performance, since they are based on the well-known static figure of merit zT.

We deduced in the particular case of an iron rotating disk, a naïve estimate of the average real part of the dynamic Thomson coefficient $\tau_{TH}$ in Equation (29), showing its giant enhancement due to rotation and to the chopped laser beam.

We remark that this prediction was deduced neglecting the effect due to the imaginary part $\chi^{\prime \prime }$ of the average magnetic susceptivity $\chi^{\prime }$, which could be associated, by the Fluctuation–Dissipation Theorem, to a thermal-noise contribution to Thomson coefficient and Thomson voltage fluctuations.

These experimental detectable predictions could be theoretically perturbed by statistical fluctuations associated to the Nyquist theorem, but, as recently investigated [6], it is not possible to extend the Johnson noise to non-equilibrium time-dependent framework with non-uniform bidimensional temperature profiles.

In fact, all the time-dependent measures of the variance of the squared voltage have, so far, been estimated using the linear relation [6].

(39) $$<V^{2}>≅\frac{2Rk_{B}T}{∆t}.$$

This approach predicts voltage drops proportional to the constant equilibrium temperature T, while quadratic relations in T have been deduced recently just assuming thermal conductance dominated by phonon scattering [39]. On the contrary, in our dynamical approach to thermoelectricity, chiral heat conduction is assumed to be dominated by electron scattering on two-dimensional out-of-equilibrium rotating disks and neither the stationary approach nor the relation in Equation (39) can be applied (these can be deduced only for one-dimensional samples at rest); the quadratic one, which is valid, while neglecting thermal electron conduction, cannot be applied either.

We remark that the chiral symmetry-breaking effect of our wavelike non-linear heat diffusion model is a specific signature of the existence of a dynamic Thomson effect and cannot be associated to any equilibrium thermal noise or to any stationary disorder effect, as recently shown in a recent paper investigating the Fluctuation–Dissipation Theorem in non-equilibrium steady states of quantum Hall liquids [40].

We think that the predicted chiral symmetry-breaking effects associated to the dynamic Thomson voltage of Equation (30) are stable with respect to Gaussian and non-Gaussian stochastic fluctuations, since this is distinct from recent models based on the Levy distributed thermal noise of Josephson junctions [41,42], and giant thermoelectric effects in ferromagnetic superconducting junctions [43]. In fact, dynamic thermal management involving dynamic tunable local magnetic phase transitions depends on harmonic thermal fields and not on local metastable states, as conventionally assumed in spin glass systems, too. Anyway, it would be important to test indirectly dynamic chiral thermoelectric effects by measuring the heat torque transfer associated to the chiral thermal emission associated to the chiral thermal Poynting vector ${\vec{P}}_{T}(T,\Omega )$ of Equation (32), for example, by measuring anisotropic optical activity due to the dynamic chiral magnetic effect [44], or by observing a rotation-induced analogue of a nonlinear chiral thermoelectric Hall effect, as recently observed with tellurium samples [45].

We outline that the predicted dynamic chiral thermal emissivity introduced in Equation (30), when confirmed experimentally, would prove the existence of a dynamical heat torque transfer due to the strong linear coupling between chiral polarized thermal emission and wavelike chiral heat diffusion on the out-of-equilibrium rotating disks.

We hope that the prediction of angular momentum-controlled chiral heat transport on rotating iron disks associated to the dynamic chiral Thomson effect will stimulate researchers to investigate the role of rotation-induced spin orbit interaction on spin-dependent electron scattering in chiral thermal conduction and in chiral thermoelectricity, extending an approach recently discussed in spintronics [46].

Finally, we expect that the hypothesis of a chiral polarized thermal emission on out-of-equilibrium rotating conductors might find interesting technological applications linking research on thermal diodes to work on non-reciprocal thermal conduction and on non-reciprocal and non-linear nanophotonics.

## 6. Conclusions

We have illustrated in this work a new dynamic chiral Thomson effect self-induced on rotating conductors exposed to a chopped laser beam, assuming the existence of a rotational nonlinear thermal Hall effect due to an out-of-equilibrium Barnett magnetic field. We showed that this new framework allows for the implementation of a novel rotational chiral approach to thermal management associated with structured helical thermal waves transporting angular momentum. We proved the existence of a dynamic chiral Thomson voltage which can be used to enhance dynamically magnetic phase transitions and to improve the performance of rotating thermoelectric devices.

We showed, finally, that this novel dynamic chiral Thomson effect is associated with a gauge-breaking thermal Poynting vector, leading to a chiral dynamic tunable thermal emissivity which, we hope, might be exploited in the future to develop a new chiral non-linear approach to nonreciprocal photonics and to chiral thermal control of magnetic storage on rotating chiral magnetic nanodevices.

## Data Availability

Data are contained within the article.

## Appendix A. Helical Chiral Thermal Fields, Non-Linear Wavelike Heat Diffusion and Dynamic Chiral Thermal Management

The non-linear dynamic approach proposed for thermoelectricity depends on the existence of the thermal fields T propagating on rotating conductors exposed to a chopped laser beam. The non-linear heat diffusion is a generalized Cattaneo–Vernotte anisotropic model, due to the self-induced Righi–Leduc effect, as given by the equations. Therefore, the new non-linear anisotropic heat transport process will depend on the out-of-equilibrium Barnett field of Equation (1) and it will be described by the non-linear heat flux law:

(A1) $$\vec{q\left(t+\tau \right)}=-k\nabla T+b\left(T\right)\vec{\Omega }\times \nabla T,$$

Unlike the conventional analysis of the thermal Hall effect, the deflection angle of the transverse heat flux is a dynamic deflection angle dependent on the thermal field T that, in polar coordinates [14], is given by

(A2) $$\theta_{R-L}(T,\Omega )=Re\frac{k_{\theta }(T)\nabla_{\theta }T}{k_{r}\left(T\right)\nabla_{r}T},$$

where on the right-hand-side we have the real part of the complex-valued functions, and with the polar and azimuthal gradients given by

(A3) $$\nabla_{r}T=\frac{dT}{dr};\;\nabla_{\theta }T=\frac{dT}{rd\theta }.$$

We will assume in the following a frequency shift due to a rotational Doppler effect of the thermal field T due to a thermal analogue of the Zel’dovich effect [25,26].

(A4) $$\frac{dT}{dt}=D_{t}T=-i\omega^{\prime }T,$$

with a rotational Doppler-shifted pulsation of the thermal field T.

(A5) $$\omega^{\prime }=\omega -m\Omega ,$$

and *m* an integer number associated to the angular momentum transferred by the chiral helical thermal field, the chirality of which depends on its sign. We note that the time derivative of Equation (A8) is a convective time derivative comoving with the rotating disk:

(A6) $$D_{t}=\frac{\partial }{\partial t}+\Omega \cdot \frac{\partial }{\partial \theta },$$

Therefore, taking the time derivative of the angle of deflection defined by (A9), it is possible to introduce a thermal Hall angular velocity $\Omega_{R-L}\left(T,\Omega \right)$ proportional to this shifted pulsation $\omega^{\prime }$

(A7) $$\Omega_{R-L}\left(T,\Omega \right)=\frac{d\theta_{R-H}(T,\Omega )}{dt},$$

with

(A8) $$\frac{d\theta_{R-H}(T,\Omega )}{dt}=Re\frac{d\theta_{R-H}(T,\Omega )}{dT}\frac{DT}{D_{t}}=\frac{d\theta_{R-H}\left(T,\Omega \right)}{dT}\omega^{\prime }Re(iT),$$

which changes sign when the shifted pulsation $\omega^{\prime }$ occurs and is zero if the resonant condition of Zel’dovich rotational superradiance is satisfied $\omega^{\prime }=0$, that is, if

(A9) ω=mΩ,

We note that this rotation-induced chiral thermal control can be tuned via the Zel’dovich effect, changing the angular velocity Ω of the disk, and, as we will see in the next paragraph, this can be associated to peaks of the Thomson coefficient and to symmetry-breaking chiral magnetic phase transitions.

In fact, it is possible to show [14] that the new thermal fields *T* satisfy, far from the focus of the laser beam incident on an ultrathin disk, a homogeneous generalized telegraphist wave equation, which generalizes the well-known Cattaneo–Vernotte model by introducing on the rotating disk the convective time derivative of Equation (2), which can be written

(A10) $${D_{t}}^{2}T+\frac{D_{t}T}{\tau \left(r,\omega^{\prime }\right)}-{v_{T}}^{2}\nabla^{2}T=0$$

We remark that our dynamic approach to thermoelectricity is based on the introduction of a Seebeck coefficient S(T), which requires that the deterministic out-of-equilibrium thermal fields T and thermal fluctuations due to stationary stochastic effects and thermal noise are averaged out.

We will find the local relaxation time $\tau \left(r,\omega^{\prime }\right)$ by looking for temporally periodic and spatially attenuated particular solutions (SATP solutions) given in polar coordinates (r,θ) by

(A11) $$T\left(r,\theta ,t\right)=T_{0}e^{i\left[\beta \left(r\right)r+m\theta -\omega^{\prime }t\right]},$$

where $T_{0}$ is the average environmental temperature and $\beta \left(r\right)$ a local complex-valued wave vector the real part of which is a solution of the differential equation in the r variable

(A12) $$Re\frac{d(\beta \left(r\right)r)}{dr}=\frac{R\omega^{\prime }}{rv_{T}}-\frac{\gamma \omega^{\prime }r}{2k},$$

where *k* is the thermal conductivity and γ the specific heat, at constant pressure, of the disk at rest.

These new, structured chiral thermal fields have, as distinct from the conventional one, a local tunable phase velocity $v_{T}$ given by

(A13) $${v^{\prime }}_{T}=\sqrt{\frac{k}{\gamma \tau (r,\omega^{\prime })}}=\frac{v_{T}}{n(r,\omega^{\prime })},$$

with the phase velocity at rest $v_{T}$ on the border of the disk and the effective index of refraction of thermal waves defined, respectively, $n(r,\omega^{\prime })$ as

(A14) $$v_{T}=\frac{2k}{\gamma R},$$

and

(A15) $$\tau \left(r,\omega^{\prime }\right)={n^{2}\tau }_{0}.$$

Taking in account Equations (12)–(15) it is possible to write the local electron relaxation time $\tau \left(r,\omega^{\prime }\right)$ as

(A16) $$\tau \left(r,\omega^{\prime }\right)=\frac{k}{\gamma {\omega^{\prime }}^{2}}\left[{{\omega^{\prime }}^{2}\left(\frac{R}{rv_{T}}-\frac{\gamma }{2k}r\right)}^{2}+\frac{m^{2}}{r^{2}}\right],$$

We note that the tunable thermal phase velocity of Equation (13) becomes linearly dependent on the rotational Doppler frequency shift $\omega^{\prime }$ on the border of the disk

(A17) $${v^{\prime }}_{T}\left(R\right)=\frac{R\omega^{\prime }}{m}=\frac{R(\omega -m\Omega )}{m},$$

which, going to zero when $\omega^{\prime }=0,$ can be exploited to prove existence of the thermal rotational super radiant effect by measuring heat transport arrest on the border of the disk.

In fact, the thermal wave phase velocity Equation (16), depending on the local relaxation time $\tau \left(r,\omega^{\prime }\right)$, and depending on the effective local thermal refractive index *n*, makes the disk an effective dispersive medium in a way similar to what has recently been proposed in a study on the hyperbolic propagation of heat on metamaterials [27].

It is possible to show [9] that the nonlinear telegraphist equation with particular solutions, Equation (16), produces determinations that are singular at the center of the disk, and have chiral isothermal helical wavefront profiles:

(A18) $$\theta \left(r\right)=\frac{R\omega^{\prime }}{mv_{T}}ln\frac{r}{R}-\frac{\gamma \omega^{\prime }}{4km}r^{2},$$

with R the disk radius, $v_{T}$ the phase velocity of the conductor at rest and with m associated to the angular momentum of the polarized laser beam considered to be transported by the helical thermal wave.

It is possible to test experimentally the rotation-induced thermal control predicted by our model by introducing a new parameter given by the difference between the angle of the isothermal profile on the border R of thin rotating disks and those of identical disks at rest, as given by

(A19) $$∆\theta \left(R,\Omega \right)=\frac{\gamma R^{2}\Omega }{4k}.$$

For example, assuming that the rotating disk is iron, inserting the values of its thermal conductivity and specific heat and choosing angular velocity Ω = 100 Hz and R = 0.01 m, from Equation (19) we have an experimentally detectable estimate of the detectable relative angle of deviation induced by rotation given by

(A20) $$∆\theta ≅\frac{4.6}{3.2}10^{-2}\;rad≅0.014\;rad≅0.8{}^{\circ}$$

which can be assumed to be a realistic detectable estimate of the transverse heat flux deviation on the timescale necessary for the helical thermal wave to reach the border of the rotating disk before being reflected backward by it.

This angle of relative thermal deflection induced by rotation could be easily tested in laboratories by using IR thermal cameras with the lock-in thermography technique to map isothermal profiles of the helical thermal waves; whenever confirmed, this could pave the way to dynamic chiral thermal management.
